# Differential Evolutionary Wiring of the Tyrosine Kinase Btk

**DOI:** 10.1371/journal.pone.0035640

**Published:** 2012-05-04

**Authors:** Hossain M. Nawaz, Per Kylsten, Noriko Hamada, Daisuke Yamamoto, C. I. Edvard Smith, Jessica M. Lindvall

**Affiliations:** 1 Clinical Research Center, Department of Laboratory Medicine, Karolinska Institutet, Karolinska University Hospital Huddinge, Huddinge, Stockholm, Sweden; 2 Europaskolan, Strängnäs, Sweden; 3 Division of Neurogenetics, Tohoku University Graduate School of Life Sciences, Sendai, Japan; 4 Bioinformatics and Expression Analysis, Department of Biosciences and Nutrition, Karolinska Institutet, Karolinska University Hospital Huddinge, Huddinge, Stockholm, Sweden; 5 BioinformaticService, Saltsjö-Boo, Sweden; University of Nottingham, United Kingdom

## Abstract

**Background:**

A central question within biology is how intracellular signaling pathways are maintained throughout evolution. *Btk29A* is considered to be the fly-homolog of the mammalian Bruton’s tyrosine kinase (Btk), which is a non-receptor tyrosine-kinase of the Tec-family. In mammalian cells, there is a single transcript splice-form and the corresponding Btk-protein plays an important role for B-lymphocyte development with alterations within the human *BTK* gene causing the immunodeficiency disease X-linked agammaglobulinemia in man and a related disorder in mice. In contrast, the *Drosophila Btk29A* locus encodes two splice-variants, where the type 2-form is the more related to the mammalian *Btk* gene product displaying more than 80% homology. In *Drosophila*, *Btk29A* displays a dynamic pattern of expression through the embryonic to adult stages. Complete loss-of-function of both splice-forms is lethal, whereas selective absence of the type 2-form reduces the adult lifespan of the fly and causes developmental abnormalities in male genitalia.

**Methodology/Principal Findings:**

Out of 7004–7979 transcripts expressed in the four sample groups, 5587 (70–79%) were found in all four tissues and strains. Here, we investigated the role of *Btk29A* type 2 on a transcriptomic level in larval CNS and adult heads. We used samples either selectively defective in *Btk29A* type 2 (*Btk29A* ^ficP^) or revertant flies with restored *Btk29A* type 2-function (*Btk29A* ^(fic Exc1–16)^). The whole transcriptomic profile for the different sample groups revealed Gene Ontology patterns reflecting lifespan abnormalities in adult head neuronal tissue, but not in larvae.

**Conclusions:**

In the *Btk29A* type 2-deficient strains there was no significant overlap between transcriptomic alterations in adult heads and larvae neuronal tissue, respectively. Moreover, there was no significant overlap of the transcriptomic changes between flies and mammals, suggesting that the evolutionary conservation is confined to components of the proximal signaling, whereas the corresponding, downstream transcriptional regulation has been differentially wired.

## Introduction

The evolution of gene expression is considered to mainly result from regulatory, rather than coding, mutations causing phenotypic differences [1]. Analyzing six different organs from ten different species it was recently reported that the rate of gene expression evolution varies among organs, lineages and chromosomes [2]. As gene products commonly function together in distinct combinations to fulfill specific tasks, concerted expression changes of selected genes may be relevant to the survival of the species. Along these lines of arguments, Brawand *et al.* described sets of different organ-specific modules, which were evolutionarily conserved [2]. In this study we have investigated the evolution of tyrosine kinase-based signaling, focusing on Bruton’s tyrosine kinase (BTK) in particular. While it is known that elements in proximal BTK-signaling are conserved even among distantly related species, it remains an open question as to whether this is also true for the entire pathways down to the effector level. Here we address this question at the transcriptomic level.

The sequence of Btk has been conserved throughout evolution, with an ancestor emerging already prior to the evolution of metazoans [3]. This kinase belongs to the Tec family of non-receptor tyrosine kinases (TFKs). While insects have only a single TFK, in vertebrates there are several kinase species, which have evolved through gene duplications. The fly kinase is most homologous to vertebrate Btk. However, in spite of the high degree of sequence conservation, the functional role of Btk seems to vary throughout evolution. In higher organisms, such as humans and other mammals, the significance of Btk lies in its function for a normal development of the immune system [4].

In the absence of mammalian Btk, B-cell receptor signaling is insufficient for the generation of mature B-lymphocytes [5], [6], [7], resulting in the immunodeficiency disease X-linked agammaglobulinemia (XLA) in humans [8], [9] and X-linked immunodeficiency disease (*Xid*) in mice [10], [11]. Insects, like *Drosophila*, possess neither B- nor T-cells. An orthologous function for *Drosophila* Btk, i.e., regulating B-cell maturation, can therefore not be expected. The *Drosophila Btk29A* locus produces two different gene products, type 1 and type 2, respectively, by differential splicing. The type 2 form reveals the highest homology to human BTK among mammalian TFKs [12]. Thus, this variant is considered to be the fly homolog of Btk by means of protein sequence [3], [13]. It is specifically required for longevity and for development of male genitalia in the fly [12].

The type 1 splice variant is shorter at the N-terminus and is unique to flies [3], [12]. The *Drosophila Btk29A* locus displays a dynamic pattern of expression through the embryonic to adult stages [FlyAtlas. http://130.209.54.32/atlas/atlas.cgi [14]. The *Btk29A* ^ficP^ is a unique allele in that it is devoid of transcription of the type 2 isoform, while leaving the type 1 isoform intact. *Btk29A* types 1 and 2 are both expressed in the central nervous system (CNS) and in the imaginal discs [12], which are epidermal thickenings in the larvae containing ecto- and mesodermal cells, which give rise to the adult organs during metamorphosis. Complete loss of function of the gene (i.e., loss of both types 1 and 2) in female germline cells, produced by using the dominant-female-sterile, FLP/FRT technique, results in oocyte undergrowth and subsequent embryonic death accompanied by defective head involution [15], [16], [17], [18], [19]. Offspring with selective loss of the type 2 transcript are viable, developing malformed male genitalia and a reduced adult life span [12]. Thus, the *Btk29A* locus exerts pleiotropic functions both through distinct spatio-temporal riming of expression as well as the generation of distinct forms of protein products by alternative splicing in various tissues. When *Btk29A* function is lost in a *Src64* mutant background, cellularization becomes incomplete in the blastoderm-stage embryo [20] and late-staged embryos fail to complete dorsal closure [21]. In *Btk29A* mutant females, oogenesis is underdeveloped presumably due to deficits in the formation of ring canals that transfer cytoplasm from nurse cells to oocytes [15], [16], [22]. Both the cellularization and oocyte phenotypes appear to result from failure to activate actin–myosin contractions [20]. Chandrasekaran and Beckendorf *et al.* have shown that *Btk29A* controls both the actin cytoskeleton and the cell cycle in the morphogenesis of embryonic salivary glands [23]. Interactions between mammalian Btk and actin have also been reported in several settings [24], [25], [26], [27], [28], suggesting this to be a common denominator in the proximal part of the Btk-signaling pathway, i.e. proximal of the Btk-dependent transcriptional regulation.

In the present study we adopted a genome-wide approach to identify Btk-dependent targets in neuronal tissues by exploring the transcriptional output from Btk-deficient and wild-type tissues, for two developmental stages in *Drosophila melanogaster*, respectively. Genes identified in this way could be *direct* or *indirect* targets for Btk-regulated transcription and outline part of the transcriptomic role of Btk in the development of the fly. The identification of Btk targets, corroborated by statistical analyses and gene set enrichment analyses, reveals parts of the scope and complexity, which Btk plays in the fly. We also conclude that there is no significant functional transcriptomic conservation for Btk targets between mouse B-cells and neuronal tissue from *Drosophila*.

**Figure 1:**
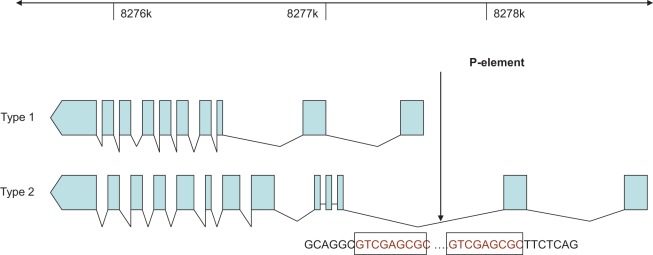
P-element insertion point detection: Schematic figure showing P-element transposon P(BmΔ-w) insertion site. Sequencing results shows P-element insertion site (arrow) within the genomic DNA of *Btk29A* at 2L: 8277721. GTCGAGCGC repeats can be seen at both end of the P insertion, which is a characteristic mark of transposon insertion. This information was further use to distinguish between mutant and revertant flies. In mutant flies this breakpoint was easily detected in amplicons (Materials and Methods), whereas the revertant did not show any P insertion.

**Figure 2:**
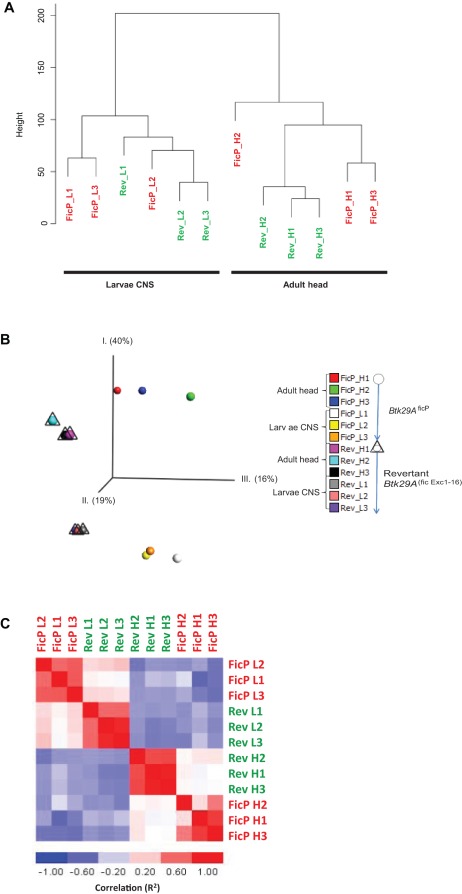
Global patterns of gene expression differences among sample groups: *A*) ***Hierarchical clustering:*** Neighbor joining tree based on pairwise distance matrices (1−ρ, Spearman correlation coefficient) for the different sample groups within this study. Sample groups fall out based on the strongest factor, larval CNS or adult head, respectively, i.e. developmental stage. Thereafter on either *Btk29A* ^ficP^ (red) or revertant (*Btk29A* ^(fic Exc1–16)^) (green). ***B*** *)* ***Factorial map of the principal-component analysis:*** The replicates within each sample collection are grouped together based on the individual samples whole genome expression profile. Revertant is denoted with Δ and ficP with a o. All samples are colored individually. The proportion of the variance explained by the principal components (axis) is indicated in parentheses in the graph. ***C*** *)* ***Sample correlation matrix:*** Spearman correlation (R^2^) is calculated and visualized by color (red-blue) in the matrix. Within the replicates for the individual sample groups the correlation is higher than between the sample groups. Also, the correlation within developmental stage was found to be higher compared to between mutant strains. Sample names are color-coded with red (*Btk29A* ^ficP^) and green (revertant).

## Results and Discussion

We performed transcriptional profiling of the central nervous system (CNS) tissues from mutant (*Btk29A* ^ficP^) and revertant (*Btk29A* ^fic exe1–16^) adults and larvae using the Affymetrix *Drosophila* Genome 2 chips, with 18,880 probe sets covering around 13,500 genes. This analysis yielded a list of affected genes known to function in longevity and aging, two biological processes impaired in *Btk29A* ^ficP^ mutants, thus validating the experimental rationale and setup. Although the phenotype-genotype association in the *Drosophila Btk29A* locus has been studied in some detail, the components and regulators of Btk29A signaling remain unexplored on the global transcriptomic level. Also, the biological processes triggered by Btk signaling defects in *Drosophila* are less well understood. Moreover, to our knowledge, the comparison of changes in gene expression profiles between Btk mutants of different animal species has not been performed before. Here, we use the strength of the *Drosophila* system in order to identify candidate effectors that take part in the Btk signaling process and use these data to perform an inter-species comparison of Btk-dependent components between mouse and fruit fly. To achieve this we have made use of the *Btk29A* type 2 mutant (*Btk29A* ^ficP^) and a revertant strain (*Btk29A* ^(fic Exc1–16)^) where wild type gene function has been restored by a jump-out event of the mutagenic P-element from the *Btk29A* ^ficP^ chromosome. This revertant fly is considered to be the most accurate wild type control for the mutant as wild-type development and life expectancy are fully restored and with the exception of the P-element, the *Btk29A*-carrying chromosome is the same for the two strains [13].

**Figure 3:**
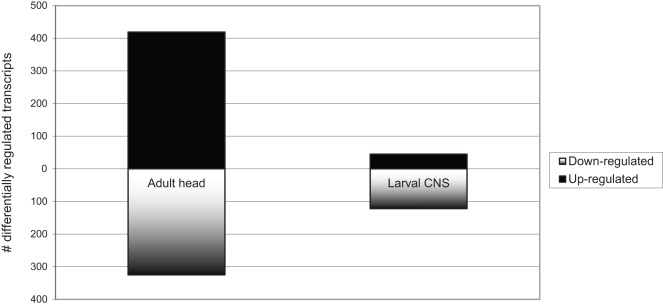
Number of differentially expressed transcripts in Btk defective flies: A differential fold-change cut-off of the Signal Log Ratio (SLR) >1,2 (difference of means between *Btk29A* ^ficP^ and revertant (*Btk29A* ^(fic Exc1–16)^) was applied to define genes whose expression was significantly different from that of the revertant and *Btk29A* ^ficP^. Bar-graph showing the number of up- and down-regulated transcripts, respectively, in adult head and larval CNS after the pair-wise comparison of revertant (*Btk29A* ^(fic Exc1–16)^) and *Btk29A* ^ficP^ data.

**Figure 4:**
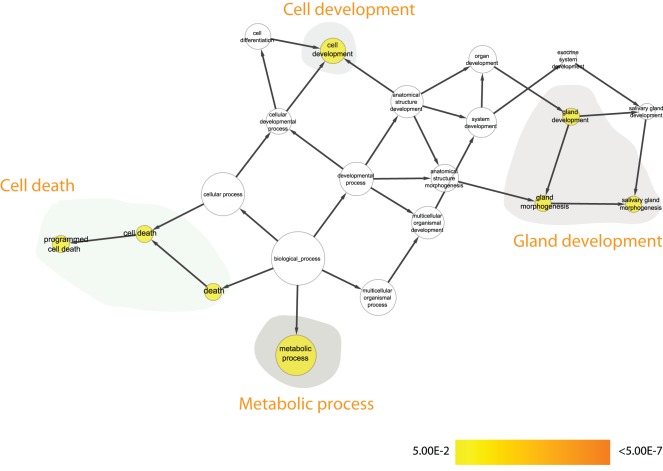
GSEA for the 391 Btk-dependent transcripts during Fly neuronal development: Enriched Biological Process clusters within the list of 391-transcripts (Btk-dependent transcripts during fly neuronal development). Figure 4 should be statistically interpreted as follows: The nodes, corresponding to different Gene Ontology clusters, are either not colored (white) i.e. not found with statistical power or colored in the scale yellow to orange, where yellow nodes are found with statistical significance *after* Bonferroni correction p<0.05 and orange colored nodes are found to be even more statistically significant after correction, with a p<7*10^−8^. The grey-zoned data highlights statistically enriched clusters of nodes (genes/transcripts), which all are represented under the manually designated heading e.g. ‘Gland development’ or ‘Cell death’. Due to space limitations in the main figure (Figure 4) we are not able to list the genes belonging to each grey-zone and cluster. This information is instead found in the Figure S1.

### Verifying P-element Insertion Point and the Nature of the Reversion

In order to define molecularly the experimental flies, we initially determined the exact location of the P-element insertion. For this we made use of P-element-specific primers (directed outwards from both the 5′ and 3′-ends of the P-element) for *Btk29A* ^ficP^ and eight primers ∼500 bp apart, covering in total a stretch of 4 kb along the genomic sequence of the *Btk29A* locus. Using PCR, amplicons were detected for both ends of the P-element. The PCR products were sequenced and the results showed that the P-element point-of-insertion into the genomic sequence was at nucleotide 2L: 8,277,721 (Figure 1). This indicates that the P-element sits within the *Btk29A* locus, although its location deviates for 866 bp from that given in FlyBase (http://flybase.org/) showing 2L:8,276,855.8,280,039 [-]. The relevant genomic primer pair produced a PCR-product from *Btk29A* ^fic exe 1–16^ revertant genomic DNA, which upon sequencing showed the P-element to having left the locus by perfect excision, leaving only the wild-type genomic sequence (data not shown).

### Transcriptional Profiling

The transcriptional profiles were analyzed with Affymetrix whole genome arrays (GeneChip *Drosophila* Genome 2.0) by a comparative approach between the “mutant” and “revertant” sample groups for either larvae or adult heads. Thus, in total, 4 different sample groups were collected (Figure 2A–C). To reconstruct strain and tissue trends in a global transcriptomic detail, we built an expression distance matrix for the four sample groups with its replicas and reconstructed a gene expression tree (Figure 2A). The tree is highly consistent with the expectation that the predominant factor to characterize the profile is the tissue type/developmental stage followed by the Btk-genotype. A quality measure for the data input is that the majority of replicates fall within the respective sample group (Figure 2A–C). To obtain an initial overview of the transcriptional expression patterns, we performed a principal-component analysis, which clearly separates the data according to sample group (Figure 2B). Figure 2C represents a Pearson correlation (R^2^) matrix for the whole transcriptomic profile for all samples included in the study. Here we see a higher intra-tissue correlation between revertant and *Btk29A* ^ficP^ mutant of the same developmental stage compared to the intra-strain correlation between the two stages/tissues (Figure 2C).

**Figure 5:**
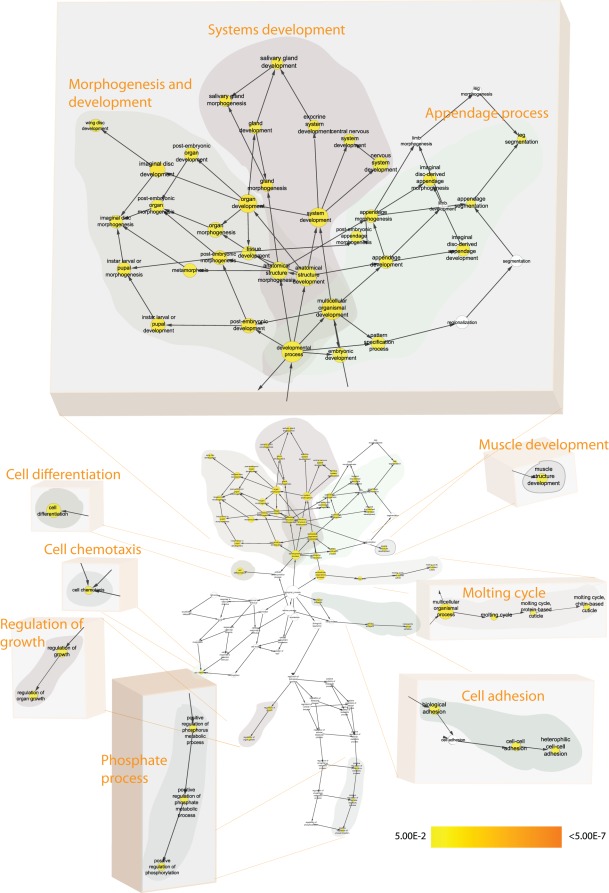
GSEA for the 167 Btk-dependent transcripts found in larvae CNS: Differentially expressed transcripts (167) were subjected to GSEA and enriched clusters were found. Figure 5 should be statistically interpreted as follows: The nodes, corresponding to different Gene Ontology clusters, are either not colored (white) i.e. not found with statistical power or colored in the scale yellow to orange, where yellow nodes are found with statistical significance *after* Bonferroni correction p<0.05 and orange colored nodes are found to be even more statistically significant after correction, with a p<7*10^−8^. The grey-zoned data highlights statistically enriched clusters of nodes (genes/transcripts), which all are represented under the manually designated heading e.g. ‘Systems development’ or ‘Regulation of growth’. Due to space limitations in the main figure (Figure 5) we are not able to list the genes belonging to each grey-zone and cluster. This information is instead found in the Figure S2.

Under such circumstances, several mathematical approaches are possible in extracting the genes that behave differently according to sample groups. When applying an ANOVA filtering in the comparison between the mutant (*Btk29A* ^ficP^) and revertant (*Btk29A* ^(fic Exc1–16)^) results, irrespective of the stage/tissue (larvae CNS or adult head) using a *p*-value of 0.05, we found 523 transcripts being statistically different between the *Btk29A* ^ficP^ and the revertant. On the other hand, when considering the stage/tissue as the decisive factor (regardless of using *Btk29A* ^ficP^ or revertant data) we detected 4489 transcripts being statistically different between the groups. This indicates, as expected, that the difference between tissue types or developmental stages in the fly gives a stronger influence on the transcriptome compared to the influence of the *Btk29A* ^ficP^ mutation. On the other hand, when *both* the tissue type (larvae CNS and adult head) *and* genotype (*Btk29A* ^ficP^ and revertant) are considered as the decisive ANOVA factors with a *p*-value <0.05 we find 391 transcripts being statistically different between the 4 sample groups. Thus, on a transcriptomic level there are 391 transcripts that, by these criteria, are Btk-dependent in *Drosophila* neuronal tissue development, from larvae to adult flies.

A differential fold-change cut-off of Signal Log Ratio (SLR) >1,2 (difference of means between FicP and revertant) was applied to define genes whose expression was significantly different between the revertant and *Btk29A* ^ficP^. The number of transcripts found to be differentially expressed between the two genotypes was more than 4 times higher in adult heads (744) as compared to larval CNS (167; Figure 3). This suggests that there are more Btk-dependent transcripts in the head, perhaps also reflecting the fact that the head is not only composed of neuronal tissue. The distribution of up- and down-regulated transcripts was approximately 50% in adult heads. In the larval CNS sample group, the distribution of differentially expressed genes/transcripts was approximately 30% up-regulated versus 70% down-regulated, suggesting that transcriptional loss-of-function is the predominating feature in the larval CNS of *Btk29A* ^ficP^ mutants.

**Figure 6:**
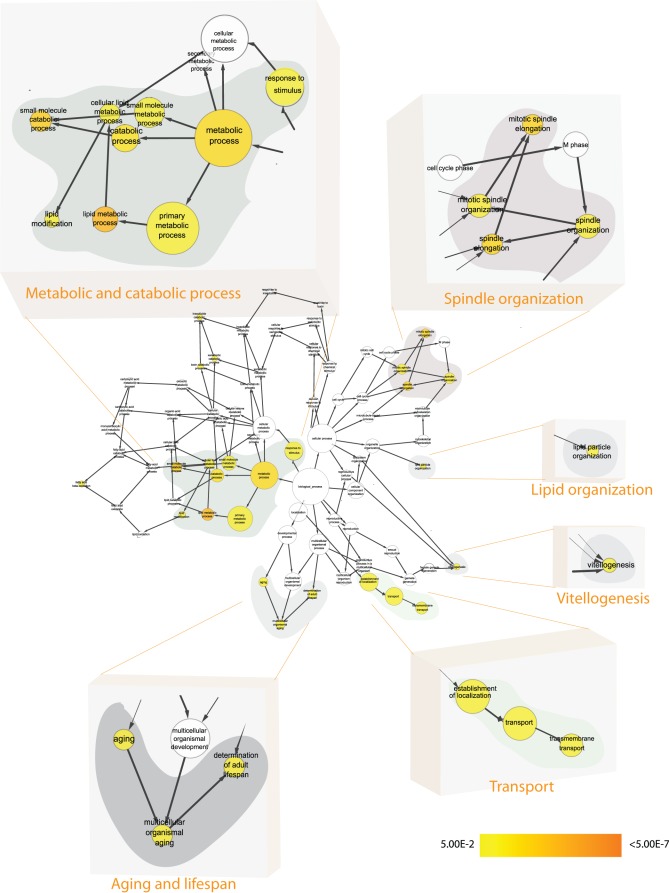
GSEA for the 744 Btk-dependent transcripts found in adult head tissue: Enriched clusters of Biological Processes were found in the Btk-dependent Adult head tissue analysis and were statistically significant. Figure 6 should be statistically interpreted as follows: The nodes, corresponding to different Gene Ontology clusters, are either not colored (white) i.e. not found with statistical power or colored in the scale yellow to orange, where yellow nodes are found with statistical significance *after* Bonferroni correction p<0.05 and orange colored nodes are found to be even more statistically significant after correction, with a p<7*10^−8^. The grey-zoned data highlights statistically enriched clusters of nodes (genes/transcripts), which all are represented under the manually designated heading e.g. ‘Transport’ or ‘Aging and life span’. Due to space limitations in the main figure (Figure 6) we are not able to list the genes belonging to each grey-zone and cluster. This information is instead found in the Figure S3.

**Table 1:** 25 genes found to overlap between *D. melanogaster* larvae CNS and adult head.

*D.melanogaster*	FlyBase ID	Differentially regulated	
Gene symbol		*Btk29A* ^ficP^	*Btk29A* ^ficP^
		Adult Head	Larvae CNS
w	FBgn0003996	4.37	3.49
TpnC47D	FBgn0010423	2.22	2.89
Α-Est1	FBgn0015568	1.83	2.21
l(3)mbn	FBgn0002440	1.39	2.18
CG5597	FBgn0034920	1.36	2.12
CG5023	FBgn0038774	1.28	1.62
CG11807	FBgn0033996	2.92	1.59
CG4398	FBgn0034126	1.91	1.53
pnt	FBgn0003118	1.4	1.31
CG2177	FBgn0039902	1.44	1.25
mthl3	FBgn0028956	*−2.23*	*−1.22*
pen-2	FBgn0053198	*−1.84*	*−1.25*
gdl-ORF39	FBgn0028377	*−1.23*	*−1.4*
CG14033	FBgn0046776	*−2.05*	*−1.4*
pst	FBgn0035770	*−1.59*	*−1.56*
CG6984	FBgn0034191	*−1.42*	*−1.65*
CG11671	FBgn0037562	*−2.01*	*−2*
CG42254	FBgn0259112	*−1.79*	*−2.02*
CG17264	FBgn0031490	*−1.72*	*−2.07*
CG32368	FBgn0052368	*−2.9*	*−5.88*
CG12241	FBgn0038304	1.46	*−1.29*
Dob	FBgn0030607	*−1.87*	1.61
Obp56h	FBgn0034475	*−3.52*	2.12
proPO-A1	FBgn0261362	*−1.64*	2.81
CG10176	FBgn0032682	1.22	*−1.28*

*Italics* denotes down-regulated genes.

* denotes genes differentially expressed NOT in the same regulatory direction for the larval CNS and adult head sample group.

### Clustering of Genes and Functional Enrichment

By performing gene set enrichment analysis using Cytoscape and the plug-in BiNGO we identified different Gene Ontology (GO) clusters being enriched in the different data sets. For the 391 probe sets indicated to be *Btk-dependent during fly neuronal development* we found four major Gene Ontology clusters to be enriched within this list (Figure 4 and a more detailed view is found in Figure S1 where the corresponding genes are listed to respective statistically significant nodes). Due to space limitations in the figures we were not able to enlarge all the titles of the nodes within the figure. We have instead tried to find commonalities within the grey-zone and manually put a ‘heading’ for each of these zones in order to give the reader an overview of the result of the GSEA. In order to see the node titles (corresponding to Gene Ontology Biological Process names) the reader can zoom in on the figure and by this be able to read the text. Amongst these clusters representing Death, Cell development, Metabolic process and Gland development we find genes previously linked to Btk function and fly development, thus validating the approach of our study, but also genes not previously known to be associated with Btk in the fly. As such, gene set enrichment analysis suggests that the genes identified reflect a *bona fide* response of fly neuronal development to the loss of Btk.

**Figure 7:**
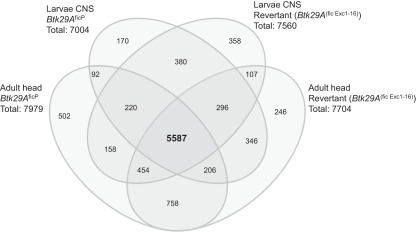
Distribution of expressed transcripts in each sample group and their overlaps: Venn- diagram showing the number of expressed probe-sets of the respective sample group and their overlap. The probe-sets found to be expressed above background (Affymetrix P-calls) in all three replicates per sample group were considered within this figure.

**Figure 8:**
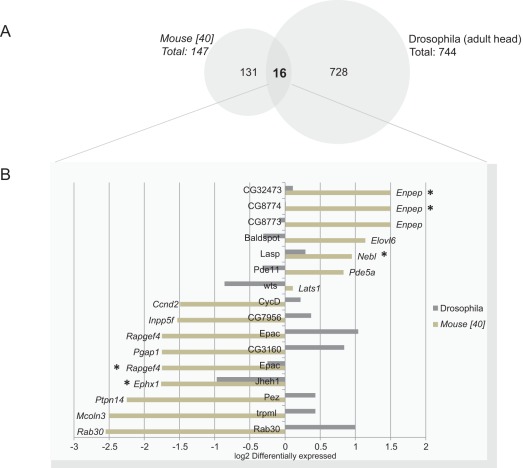
Comparison between Mouse and Drosophila Btk-dependent transcript: **A)** ***Venn-diagram:*** showing the overlap between Btk-dependent Transitional type 1 B-cells from Btk-defective mice [40] (a total of 147 differentially expressed genes) and the Btk-dependent transcripts found in *Btk29A* ^ficP^ *Drosophila* adult head (a total of 744 differentially expressed genes). **B)** ***Thirteen orthologous transcripts found in Btk KO mouse Transitional type 1 B-lymphocytes and in Btk29A defective flies.*** Bar-graph showing the 16 transcripts, corresponding to 13 genes, found to be common in a homology search between the Btk-dependent transcripts found in the *Btk KO* mouse Transitional type 1 B-cells compared to its healthy control strain [40] and the current *Drosophila Btk29A* ^ficP^ study. Out of these 13 genes, 5 genes showed the same regulatory direction i.e. being up- or down-regulated in the respective Btk-defective strain (denoted as *).

In the larval CNS we find 167 Btk-dependent transcripts being differentially expressed with an SLR >1.2 between *Btk29A* ^ficP^ and revertant flies. A Gene Set Enrichment Analysis on this set of transcripts reveals Gene Ontology terms which mirror undifferentiated progenitor cells for the future adult i.e., ‘imaginal’ cells (Figure 5 and a more detailed view is found in Figure S2 where the corresponding genes are listed and colored depending on the direction of the regulation to respective statistically significant nodes). Indeed, one of the processes showing the highest statistical score (after correction with the Benjamini-Hochberg algorithm) is ‘Imaginal disc development’ (GO:0007444′′) with a corrected p-value <0.003. Another GO-term, ‘Developmental process’ (for *D. melanogaster*), characterized by 42 genes (in our 167 gene list) out of a total of 117(in the GO ‘Developmental process’ gene list), which comprises 35.8% of the total number of genes in the GO term and 25% of the genes being differentially expressed in “larval CNS *Btk29A* ^ficP^”.

For the 744 differentially expressed (SLR>1.2) transcripts found in “adult head *Btk29A* ^ficP^” there is an overrepresentation of the terms ‘Aging’ (GO:0007568) and ‘Determination of lifespan’ (GO:0008340) (Figure 6 and a more detailed view is found in Figure S3 where the corresponding genes are listed and colored depending on the direction of the regulation to respective statistically significant nodes). These findings were expected as life span is reduced in *Btk29A* ^ficP^ mutants [12], [13]. As these Gene Ontology terms are not found to be enriched in the larval CNS tissue samples we hypothesize that the reduction of life span, due to the Btk defect, is manifested later in the life of the fly and therefore cannot be foreseen at the larval stage. There are no Gene Ontology terms found to be enriched in *both* adult heads and larval CNS from a global transcriptomical point of view. This potentially indicates that Btk plays somewhat different roles at these two developmental stages in *Drosophila*. This is further supported by the finding that the larval CNS and adult head sample groups shared only 20 transcripts (corresponding to 20 genes) out of a total of 29 transcripts (corresponding to 25 genes), whose expression was either up- or down-regulated in *Btk29A* ^ficP^ mutants at *both* developmental stages, and a half of these genes was up-regulated and another half was down-regulated (Table 1 and Table S1). Table S1 mirrors Table 1 in addition to adding the dimension of every gene’s Gene Ontology term including Gene Ontology ID. Btk might be more important in adult rather than larval neural tissues due to the finding of ‘Spindle organization’-enriched genes, which belong to the Gene Ontology term ‘mitotic spindle’. Thus, in the adult developmental stage, Btk might play a role in proliferation of the glia, since there are no neuroblasts in the adult head. On the other hand, a gene set enrichment analysis (using the web-based GO tool ‘DAVID’) performed on the above mentioned 25 genes (Table 1 and Table S1) revealed a statistical significant overrepresentation of the GO term ‘Behavior’ (GO:0007610), indicating that the Btk defect, independent of the developmental stage, might be manifested via the biological process ‘Behavior’ in *Drosophila*. Another interesting observation throughout the gene set enrichment analysis was that the number of down-regulated transcripts predominates independent of data set introduced, mirroring the loss-of-function nature of *Btk29A* ^ficP^ mutants.

### Transcriptional Comparison between Mouse and Fruit fly Btk-defective Cells

A central question in biology is to what level protein function in intracellular signaling pathways is conserved through evolution of species, like e.g. between mammals and insects. For instance, it has been proven possible to ‘humanize’ the fly by introducing human genes of interest, including the human *BTK* gene, and studying them in an organotypic context [13], [29], [30]. Previous studies on components in the JAK/STAT signaling pathway has revealed a small, but statistically significant, overlap between *Drosophila* and mammals at a transcriptomic level [31]. In sea urchins and sea stars, organisms that diverged from their common ancestor 500 million years ago, a three-gene feedback loop involving Notch-signaling controls endoderm and mesoderm development in both overlapping and distinct ways [32]. Furthermore, appendages of different insects show divergent use of developmental regulatory genes, including the helix-loop-helix, homeodomain transcription factor Distal-less [33]. These phenomena have been referred to as gene regulatory network “plug-ins”, in which sub-circuits are frequently re-deployed during evolution while the internal structure remains the same [34]. Further example of such rewiring comes from protein kinase A (PKA) catalytic subunit signaling in the fungus of the genus, *Cryptococcus* [35]; two sibling species of this pathogen express two different catalytic subunits of PKA, and alternative subunits are used in virulence factor production and mating in each species. It is envisaged that an ancestral PKA underwent a duplication event leading to the two catalytic subunit genes, one of which retained its function for the given biological processes in each species. Whether the “unused” subunit has undergone neofunctionalization with a novel gain-of-function for another biological activity is not known, but this example demonstrates evolutionary reconfiguration of a signaling cascade. Recently, rewiring of both prokaryotic and eukaryotic signaling pathways has been achieved using rational design, demonstrating another aspect of the alteration of signal transduction pathways [36], [37].

Apart from the well-known developmental role in the immune system, mammalian Btk has been shown to exert two counteracting roles in apoptosis, one as a protector and in the other as an inducer of apoptosis depending on the context [38], [39]. This reflects the diverse role of a protein within a species. In the fruit fly, loss of the *Btk29A* type 2 transcript is compatible with life, as opposed to loss of both types 1 and 2 of *Btk29A*, which is embryonic lethal. However, type 2 mutant flies display reduced life span as well as malformation of the male genitalia [15], [16]. We have previously published work on Btk-defective mouse B-lymphocytes using gene expression profiling [40], [41]. In order to identify factors with conserved functions throughout evolution we conducted an inter-species comparison of the Btk-dependent transcripts identified in *Drosophila* against our previous transcriptomic data obtained with mouse Btk-defective Transitional type 1 B-lymphocytes [40]. Figure 7 illustrates the number of transcripts found to be expressed in every *Drosophila* sample group examined and the level of overlap between the assemblies. This indicates that approximately 30% of the *Drosophila* genome is expressed at the time examined in the respective tissues and strains. In comparison to these numbers, in mammalian B-lymphocytes [40] we see that 37% of all transcripts in the mouse genome are expressed at any given time. Figure 8A shows a Venn-diagram that illustrates the overlap between the differentially expressed transcripts found in mouse Btk-defective Transitional type 1 B-cells (a total of 147 regulated genes) and the *Drosophila Btk29A* ^ficP^ adult head (a total of 744 differentially expressed transcripts). The overlap between the two species is only sixteen transcripts, corresponding to 13 genes in the Btk-defective mice found in our previous study [40] having orthologs in the Btk-dependent transcripts identified in *Drosophila* (Figure 8B). Of these 16 transcripts only 5 are found to show parallel changes in *Drosophila* and mice being either up- or down-regulated in the Btk-defective strains (denoted as * in Figure 8B). By analyzing the gene expression profile from Btk-defective flies representing two different developmental stages and comparing these to mammalian Btk-defective B-cells we conclude that there is no significant overlap in the transcriptome for Btk-defective mammalian B-cells and neuronal cells from *Drosophila*. Based on these observations, we conclude that there is no significant functional transcriptomic conservation for Btk targets between the mammals and fly species.

### Concluding Remarks

Although the upstream signaling protein components of *Btk29A* seem to be conserved throughout evolution, the downstream transcriptional pattern seems not to be comparable between the fruit flies and mice. The Btk-dependent gene expression profile seen in mouse transitional type 1 B-lymphocytes from Btk-defective animals thus differs from the global transcriptomic signature seen in *Btk29A* type 2-defective neural tissues from *Drosophila*. This is in contrast to JAK/STAT signaling in which both the upstream and downstream components were reported to be conserved [31].

Indeed, large scale profiling data must be interpreted with caution and the genes identified here await ultimate proof as to whether they represent the *bona fide* effectors of *Btk29A*-mediated developmental signaling. Although detailed mechanisms of action of individual effectors and their roles linked to *Btk29A* function remains partially unknown, it is interesting to note that a profile related to life-span was recognized, suggesting that our transcriptomical mapping approach has effectively identified different pathways and effectors likely to play roles in Btk signaling and functioning regarding fruit fly development.

## Materials and Methods

### Drosophila Melanogaster *Strains*

*w;Btk29A* ^ficP^ */CyO* and *w1118; Btk29A* ^(fic Exc1–16)/^ *SM1* were generated in the Yamamoto laboratory (http://www.jst.go.jp/erato/project/yks_P/yks_P.html). Flies were raised on standard medium on a 12∶12 h L:D cycle, at 23°C and at 55% RH. The *Btk29A* ^ficP^ chromosome was put over the *CyO, P{w[+mC] = ActGFP}JMR1* (source: Bloomington stock center) balancer using standard crossing schemes. This balancer was later used for sorting heterozygous (GFP-positive) from homozygous *Btk29A* ^ficP^ mutants (GFP-negative). *Btk29A* ^(fic Exc1–16)^ flies were kept homozygous viable in stocks.

### P-element Breakpoint Determination using PCR

Four kb (2L:8274950,8279050) surrounding the *Btk29A*-locus was used as a template to construct 8 forward-, and 8 reverse-oriented primers covering the entire 4-kb region from both ends with a 500 bp spacing. Primers were also made for the 3′- and 5′-ends of the *Btk29A* ^ficP^ -responsible P-element (BmΔ-w). Both primers were facing outwards from the P-element. PCR was performed using ABI GeneAmp™ system 2700 and the insertion site was determined by sequencing the PCR product (http://www.eurofinsdna.com ).

### Dissection and Sample Preparation

Flies were anaesthetized using CO_2_, then immediately dissected. The tissues dissected were the complete heads, severed at the neck from adult flies and the CNS (developing brain), including the optic lobes from third instar wandering stage larvae. Tissues were collected into RLT buffer, pooled and extracted for RNA using Qiagen RNeasy RNA extraction kit (Qiagen, Valencia, CA, USA). In total there were 3 replicates for *Btk29A* ^ficP^ and *Btk29A* ^(fic Exc1–16)^ sample groups (the larval CNS and adult heads).

### RNA Isolation and Microarray Processing

RNA was extracted and *in vitro* reverse-transcribed according to Affymetrix protocol. Quality assurance was provided by using an Agilent 2100 Bioanalyzer (Agilent Technologies, Palo Alto, CA, USA), after RNA extraction and *in vitro* transcription steps. *Drosophila* genome 2 expression arrays were hybridized and read using standard Affymetrix procedures. Microarrays were run at the Bionformatics and Expression Analysis core facility (http://apt.bea.ki.se/index.html) located at Karolinska Institutet, Huddinge (Novum).

### Processing of the High-throughput Arrays and Analysis

GeneChip.CEL files were analyzed by using R statistical programming language, Bioconductor (http://www.bioconductor.org/), and the affy package. Data were initially RMA normalized first across the samples and then within each sample group. RMA normalized data were then scaled to a common median value. Both raw and pre-processed data is deposited in GEO (http://www.ncbi.nlm.nih.gov/geo/) (GSE30627).

Further filtering, within sample group analyses and pair-wise comparisons were carried out using dChip (https://sites.google.com/site/dchipsoft/, http://biosun1.harvard.edu/complab/dchip/) and Microsoft Excel.

For the Venn-diagram (Figure 7), the probe-sets found to be expressed above background (Affymetrix P-calls) in all three replicates per sample group were considered within this figure.

### Comparison between Btk-dependent Transcripts from Btk KO Mouse Transitional Type 1 B-cells [40] and Adult Btk29A^ficP^ Drosophila Btk-dependent Transcripts

An inter-species comparison was conducted of the Btk-dependent transcripts identified in *Drosophila*, a total of 744 Btk-dependent transripts were found, compared to our previous transcriptomic data obtained from mouse Btk-defective Transitional type 1 B-lymphocytes [40], where a total of 147 Btk-dependent genes were reported as differentially expressed. The 147 genes found to be Btk-dependent in Btk-defective mouse Transitional type 1 B-cells were investigated for orthologs/homologs in the *Drosophila melanogaster* specie. We made use of the Affymetrix oligonucleotide array comparison to find which transcripts could be possible homologs (www.affymetrix.com/analysis/index.affx). The converted orthologs were then compared with the differentially expressed Btk-dependent transcripts found in the *Drosophila Btk29A* ^ficP^ adult heads.

### Gene Set Enrichment Analysis

Enriched GO clusters were analyzed using Cytoscape (http://www.cytoscape.org/) [26], with the plug-in system BiNGO [27] in addition to the DAVID web-tool (http://david.abcc.ncifcrf.gov/home.jsp) [28], [29]. The Hyper-geometric Test with Benjamini-Hochberg False Discovery Rate Correction was chosen for both the analyses [27].

## Supporting Information

Figure S1 **Gene Set Enrichment Analysis for Gene Ontology (biological process) clusters for the 391 Btk-dependent transcripts during Fly neuronal development (** **Figure 4** **in manuscript):** Enriched Biological Process clusters within the list of 391-transcripts (Btk-dependent transcripts during fly neuronal development). The genes belonging to respective cluster are written next to the grey-zoned areas. For the main Figure 4, Figure S1 shows the respective genes found for each cluster (grey-zoned in Figure 4).
(EPS)

Figure S2 **Gene Set Enrichment Analysis for Gene Ontology (biological process) clusters for the 167 Btk-dependent transcripts found in larvae CNS:** Differentially expressed transcripts (167) were subjected to GSEA and enriched clusters were found. The genes belonging to respective cluster are marked in either red (up-regulated) or blue (down-regulated) depending on the direction of the gene. For the main Figure 5, Figure S2 shows the respective genes found for each cluster (grey-zoned in Figure 5). The genes belonging to respective cluster are marked in either red (up-regulated) or blue (down-regulated) depending on the direction of the gene.
(EPS)

Figure S3 **Gene Set Enrichment Analysis for Gene Ontology (biological process) clusters for the 744 Btk-dependent transcripts found in adult head tissue:** Enriched clusters of Biological Processes were found in the Btk-dependent Adult head tissue analysis and were statistically significant. The genes belonging to respective cluster are marked in either red (up-regulated) or blue (down-regulated) depending on the direction of the gene. For the main Figure 6, Figure S3 shows the respective genes found for each cluster (grey-zoned in Figure 6). The genes belonging to respective cluster are marked in either red (up-regulated) or blue (down-regulated) depending on the direction of the gene.
(EPS)

Table S1 Table S1 mirrors Table 1 (*25 genes found to overlap between D. melanogaster larvae CNS and adult head*) in addition of adding the dimension of every gene’s Gene Ontology term including Gene Ontology ID.
(DOCX)
