# Supplementary material for: Differential Evolutionary Wiring of the Tyrosine Kinase Btk
Source: PLoS One. 2012 May 4;7(5):e35640. doi: 10.1371/journal.pone.0035640 (PMC3344829; doi:10.1371/journal.pone.0035640)
Supplement: Table S1 — mirrors Table 1 (25 genes found to overlap between D. melanogaster larvae CNS and adult head) in addition of adding the dimension of every gene’s Gene Ontology term including Gene Ontology ID. (DOCX) [file pone.0035640.s004.docx]

**Table S1:** 25 genes found to overlap between *D. melanogaster* larvae CNS and adult head. Here with Gene Ontology descriptions and Gene Ontology IDs.

|  |  |  |  | **Differentially regulated** | |
| --- | --- | --- | --- | --- | --- |
| ***D.melanogaster***  **Gene symbol** | **FlyBase ID** | **Gene Ontology description** | **GO ID** | ***Btk29A*^ficP^**  **Adult Head** | ***Btk29A*^ficP^**  **Larvae CNS** |
| w | FBgn0003996 | cellular biogenic amine biosynthetic process | GO:0042401 | 4.37 | 3.49 |
|  |  | cGMP transport | GO:0070731 |  |  |
|  |  | compound eye pigmentation | GO:0048072 |  |  |
|  |  | male courtship behavior | GO:0008049 |  |  |
|  |  | memory | GO:0007613 |  |  |
|  |  | ommochrome biosynthetic process | GO:0006727 |  |  |
| TpnC47D | FBgn0010423 | Calcium ion binding | GO:0005509 | 2.22 | 2.89 |
| Α-Est1 | FBgn0015568 | carboxylesterase activity | GO:0004091 | 1.83 | 2.21 |
| l(3)mbn | FBgn0002440 | bristle development | GO:0022416 | 1.39 | 2.18 |
|  |  | wing disc development | GO:0035220 |  |  |
| CG5597 | FBgn0034920 | - | - | 1.36 | 2.12 |
| CG5023 | FBgn0038774 | actomyosin structure organization | GO:0031032 | 1.28 | 1.62 |
| CG11807 | FBgn0033996 | cell communication | GO:0007154 | 2.92 | 1.59 |
| CG4398 | FBgn0034126 | - | - | 1.91 | 1.53 |
| pnt | FBgn0003118 | epidermal growth factor receptor signaling pathway | GO:0007173 | 1.4 | 1.31 |
|  |  | heart development | GO:0007507 |  |  |
|  |  | heterophilic cell-cell adhesion | GO:0007157 |  |  |
|  |  | open tracheal system development | GO:0007424 |  |  |
|  |  | ovarian follicle cell development | GO:0030707 |  |  |
|  |  | peripheral nervous system development | GO:0007422 |  |  |
|  |  | R7 cell development | GO:0045467 |  |  |
|  |  | Ras protein sig-l transduction | GO:0007265 |  |  |
|  |  | regulation of transcription, D-dependent | GO:0006355 |  |  |
|  |  | secondary branching, open tracheal system | GO:0007429 |  |  |
| CG2177 | FBgn0039902 | metal ion transport | GO:0030001 | 1.44 | 1.25 |
|  |  | transmembrane transport | GO:0055085 |  |  |
| mthl3 | FBgn0028956 | determination of adult lifespan | GO:0008340 | *-2.23* | *-1.22* |
|  |  | G-protein coupled receptor protein signaling pathway | GO:0007186 |  |  |
|  |  | response to stress | GO:0006950 |  |  |
| pen-2 | FBgn0053198 | endopeptidase activity | GO:0004175 | *-1.84* | *-1.25* |
| gdl-ORF39 | FBgn0028377 | spermatogenesis | GO:0007283 | *-1.23* | *-1.4* |
| CG14033 | FBgn0046776 | - | - | *-2.05* | *-1.4* |
| pst | FBgn0035770 | learning or memory | GO:0007611 | *-1.59* | *-1.56* |
|  |  | olfactory learning | GO:0008355 |  |  |
| CG6984 | FBgn0034191 | metabolic process | GO:0008152 | *-1.42* | *-1.65* |
| CG11671 | FBgn0037562 | - | - | *-2.01* | *-2* |
| CG42254 | FBgn0259112 | - | - | *-1.79* | *-2.02* |
| CG17264 | FBgn0031490 | - | - | *-1.72* | *-2.07* |
| CG32368  * | FBgn0052368 | - | - | *-2.9* | *-5.88* |
| CG12241 | FBgn0038304 | regulation of Rab GTPase activity | GO:0032313 | 1.46 | *-1.29* |
|  |  | regulation of Ras GTPase activity | GO:0032318 |  |  |
|  |  | regulation of Rab protein signal transduction | GO:0032483 |  |  |
|  |  | regulation of GTPase activity | GO:0043087 |  |  |
|  |  | regulation of Ras protein signal transduction | GO:0046578 |  |  |
|  |  | regulation of small GTPase mediated signal transduction | GO:0051056 |  |  |
| * |  | regulation of hydrolase activity, | GO:0051336 |  |  |
| Dob  * | FBgn0030607 | lipid catabolic process | GO:0016042 | *-1.87* | 1.61 |
| Obp56h | FBgn0034475 | sensory perception | GO:0007600 | *-3.52* | 2.12 |
|  |  | sensory perception of chemical stimulus | GO:0007606 |  |  |
|  |  | sensory perception of smell | GO:0007608 |  |  |
|  |  | behavior | GO:0007610 |  |  |
|  |  | chemosensory behavior | GO:0007635 |  |  |
|  |  | response to pheromone | GO:0019236 |  |  |
|  |  | neurological system process | GO:0050877 |  |  |
|  |  | response to organic substance | GO:0010033 |  |  |
|  |  | olfactory behavior | GO:0042048 |  |  |
| * |  | cognition | GO:0050890 |  |  |
| proPO-A1 | FBgn0261362 | defense response | GO:0006952 | *-1.64* | 2.81 |
|  |  | Immune response | GO:0006955 |  |  |
|  |  | innate immune response | GO:0045087 |  |  |
| * |  | oxidation reduction | GO:0055114 |  |  |
| CG10176 | FBgn0032682 | - | - | 1.22 | *-1.28* |

*Italics* denotes down-regulated genes.

* denotes genes differentially expressed NOT in the same regulatory direction for the larval CNS and adult head sample group.
